# Incorporation of omics analyses into artificial gravity research for space exploration countermeasure development

**DOI:** 10.1007/s11306-015-0942-0

**Published:** 2016-01-20

**Authors:** Michael A. Schmidt, Thomas J. Goodwin, Ralph Pelligra

**Affiliations:** Sovaris Aerospace, LLC, Advanced Pattern Analysis & Countermeasures Group, Research Innovation Center, Colorado State University, 3185 Rampart Road, Fort Collins, CO 80521 USA; Disease Modeling and Tissue Analogues Laboratory, Biomedical Research and Environmental Sciences Division, NASA Lyndon B. Johnson Space Center, Houston, TX 77058 USA; Chief Medical Officer, NASA Ames Research Center, Moffett Field, CA USA

**Keywords:** Omics, Gravity, Artificial gravity, Space flight, Astronaut, Countermeasures

## Abstract

The next major steps in human spaceflight include flyby, orbital, and landing missions to the Moon, Mars, and near earth asteroids. The first crewed deep space mission is expected to launch in 2022, which affords less than 7 years to address the complex question of whether and how to apply artificial gravity to counter the effects of prolonged weightlessness. Various phenotypic changes are demonstrated during artificial gravity experiments. However, the molecular dynamics (genotype and molecular phenotypes) that underlie these morphological, physiological, and behavioral phenotypes are far more complex than previously understood. Thus, targeted molecular assessment of subjects under various G conditions can be expected to miss important patterns of molecular variance that inform the more general phenotypes typically being measured. Use of omics methods can help detect changes across broad molecular networks, as various G-loading paradigms are applied. This will be useful in detecting off-target, or unanticipated effects of the different gravity paradigms applied to humans or animals. Insights gained from these approaches may eventually be used to inform countermeasure development or refine the deployment of existing countermeasures. This convergence of the omics and artificial gravity research communities may be critical if we are to develop the proper artificial gravity solutions under the severely compressed timelines currently established. Thus, the omics community may offer a unique ability to accelerate discovery, provide new insights, and benefit deep space missions in ways that have not been previously considered.

## Introduction

According to NASA’s flexible deep space exploration path, the next major steps in human spaceflight include flyby and orbital missions to the Moon, Mars, near earth asteroids, lunar and Martian landings, and combinations of these scenarios (Paloski et al. 2014). The first crewed private deep space mission is expected to launch in 2022 (Inspiration Mars; Clyne 2014; Mooring 2013), followed by the Mars One Mission in 2024 (Sewell 2012). This affords less than 7 years to sufficiently answer one of the most pressing questions related to human safety and performance, and to pass these data on to spacecraft designers. That is, what is the true benefit of an *artificial gravity* (AG) regime and how do we best validate its efficacy? Answering such questions will require discovery methods that can accommodate these accelerated timelines and handle the requisite complexity that investigators will most certainly encounter.

## Background: gravity on earth and in space

The incompletely defined, but inevitable, adverse effects of 30-months exposure to reduced gravity during a Mars mission (0 G during transit and 0.38 G, while on the planetary surface) are not likely to be remedied by exercise, pharmaceuticals, or a combination of both (Paloski et al. 2014). The reasons for their failure are predictable.

Ever since the Late Devonian period, 375 million years ago, when primitive vertebrates first migrated from the buoyancy of the ancient sea onto land, all life forms on Earth have had to evolve adaptive mechanisms to contend with the unremitting and ubiquitous “force” of gravity. The complex constellation of hormonal, reflex, and central nervous system adaptive changes that enable humans to maintain a bipedal posture in three dimensional space are dependent on the presence of the gravitational acceleration vector for stimulation and orientation. When the gravitational umbilical cord is severed to allow human space travel, these adaptive mechanisms become maladaptive, leading to dysfunctional changes—particularly in the cardiovascular, musculoskeletal, and vestibular systems.

None of the present countermeasures can replace the unique features and effects of gravity, which is the missing critical ingredient. But there is growing promise that “artificial” gravity (AG) can do so. “Artificial gravity” is a tolerable misnomer that refers to the centripetal acceleration forces and resulting inertia that are imposed on a human who is exposed to continuous radial motion in a centrifuge. Although centrifugal forces during a centrifugation as the product of artificial gravity generation show large gravity gradients along their lines of action (e.g. 0.2 G in the region of the head and 2 G in the region of the calves on a short arm centrifuge when the subject is positioned in +Gz direction), centrifugation is, nevertheless, the application that comes closest to real gravity (Clément et al. 2015).

It is fortuitous for future space voyagers that, in accordance with Einstein’s ‘Theory of Equivalence,’ the human body cannot distinguish between the effects of accelerations generated by gravitation or by centrifugation (though effects of the Coriolis force must be considered). It responds identically to both, at the cellular, systemic, and behavioral levels.

Just as it is now critical for the space traveler to carry along sufficient oxygen, food, water, and ambient pressure to sustain life, AG offers the promise of a “portable” *gravity equivalent* that can prevent the harmful effects of prolonged exposure to “weightlessness” and minimize the adjustments required to return to, and function within, earth’s 1 G environment or partial gravities, such as the Moon (0.17 G) and Mars (0.38 G).

The role of AG is one of the many challenges confronting space researchers and it compels us to address at least two seminal points. First, the degree to which AG is an effective countermeasure against the detrimental effects of prolonged space flight, must be established. Second, if deemed an effective countermeasure, we must design an optimal prescription for its use. Should exposure to AG be continuous or intermittent? Should AG be deployed only during sleep or periodically throughout the day? What is the optimal duration for the application of AG? At what intensity (G load) should AG be applied for a given space condition or environment?

**Fig. 1:**
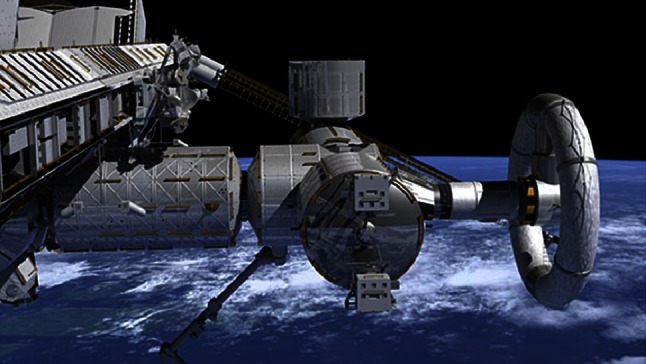
Artificial gravity rotating wheel model. The rotating wheel (*right*) affixed to the Nautilus-X spacecraft design is one conception of how artificial gravity might be applied. Understanding the scale, rate of rotation, radius length, and specific artificial gravity parameters will be central to achieving successful mission AG profiles. (Image: NASA)

The application of artificial gravity requires spinning the space vehicle (or a component thereof) during any prolonged exposure to weightlessness (Fig. 1). Recently, the Artificial Gravity Working Group (AGWG) agreed that this was needed, though the optimum parameters for such applications are not yet well defined. Moreover, answering these most fundamental questions with precision has significant engineering, biomedical, and cost implications.

NASA and private industry are exploring more than nine design reference missions (DRMs) to send humans into deep space for long-duration periods (years). AG and gravity-based research programs encompassing animal investigations on the International Space Station, and short- and long-radius centrifugation of humans on the ground are either being initiated, or have previously been conducted by NASA and the research community. This will be used to identify the specific gaps associated with possible AG profiles and perform trade-off feasibility analyses between potential AG profiles and non-AG solutions (Paloski et al. 2014; Young et al. 2009; Johnson et al. 1997). Given the compressed timeline of only 7 years to better answer key AG questions, we propose that an omics-based approach be considered for all artificial gravity research, with the intent to accelerate our understanding of the human response to AG under these compressed mission time lines.

## Understanding the molecular landscape beneath the AG phenotype

Various phenotypic changes are demonstrated during artificial gravity experiments. The general phenotypes under observation are typically characterized as morphological, physiological, and behavioral. In such studies, attention is given to a broad range of measurement methods, which frequently includes targeted molecular profiling. This approach has been informative for some decades.

**Fig. 2:**
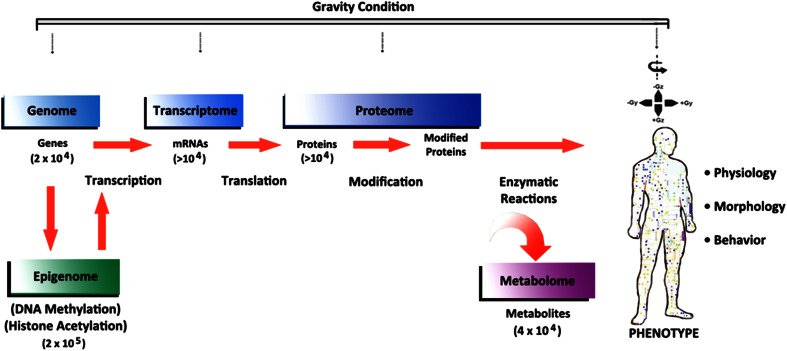
Molecular landscape that underlies the morphological, physiological, and behavioral phenotype. The progression from genome to phenotype is shown above (*left to right*), representing the extraordinary complexity of this landscape. *Numbers in parentheses* represent the estimated number of unique molecular forms found in each subdomain. Measurement in artificial gravity experiments frequently occurs at the physiological, morphological, and behavioral levels. Targeted components of the molecular landscape (limited, selected chemistry profiles) are also sometimes measured under different gravity exposure paradigms, though they represent a small snapshot of the whole. The application of omics to artificial gravity experiments today is quite limited, representing an enormous opportunity to clarify biological responses and advance the field. (Image: Sovaris Aerospace) (Adapted from Gerszten and Wang 2008)

However, the molecular dynamics (genotype and molecular phenotypes) that underlie these morphological, physiological, and behavioral phenotypes are far more complex than previously understood (Fig. 2). Thus, targeted molecular assessment of subjects under various G conditions can be expected to miss important patterns of molecular variance that inform the more general phenotypes typically being measured. To better understand this dynamic, it is useful to explore some of the artificial gravity work that exists, the status of molecular profiling to date, and the frontier of omics applications in AG research.

## Targeted molecular assessment in space and on earth

There are a limited number of study paradigms with which to study artificial gravity conditions, most of which involve centrifugation. Among the primary study paradigms are, (1) humans, animals, tissues, or cells subjected to centrifugation, while in space; (2) humans, animals, tissues, or cells on earth exposed to hypergravity conditions, which use earth gravity (1 G) as a control compared with varying degrees of elevated G forces applied through centrifugation; (3) Head up or head down bed rest studies (short, intermediate, long duration), coupled with centrifugation; 4) Limb suspension studies with centrifugation, and 5) others (Tables 1 and 2). For the purpose of this review, we will focus on the limited number of studies in humans, animals, tissues, or cells in the in-flight (0G-AG+) or Earth-based (1G-AG+) condition (Table 3).

**Table 1:** Space-based partial gravity analogues

Name of analogue	Species suitability	Exposure duration	References
Short-radius human centrifugation	Human	Acute	Clément and Slenzka (2006)
Long-radius human centrifugation	Human	Chronic	Clément et al. (2001) and Clément (2015)
Long-radius human centrifugation	Animal	Chronic	Clément and Slenzka (2006)
Rotating wall vessel (RWV)	Cells or tissue cultures	Chronic	Ruoxiang et al. (2005); Goodwin et al. (2004); Kleis et al. (2005); Becker and Souza (2013)

**Table 2:** Earth-based partial and microgravity analogues

Name of analogue	Species suitability	Exposure duration	References
Parabolic flight	Human	Acute	Pletser et al. (2012)
Head-up tilt	Human	Acute	Cavanagh et al. (2013)
Supine or head-down tilted short-radius centrifugation	Human	Acute	Clément et al. (2015); Young and Paloski (2007); Greenleaf et al. (1996)
Whole-body weighted garment water immersion	Human	Acute	Johansen et al. (1998),
Lower body positive pressure	Human	Acute	McNeill et al. (2015)
Overhead suspension	Human	Acute	Ellman et al. (2013); Norcross et al. (2010a, b, 2012)
Head-out graded water immersion	Human	Acute	Regnard et al. (2001)
Head-out graded dry immersion	Human	Chronic	Navasiolava et al. (2011)
Long-radius centrifugation (upright, supine, or head-down tilted)	Human	Acute or chronic	Graybiel et al. (1960, 1965); van Loon (2009)
Head-up bedrest	Human	Chronic	Pavy-Le Traon et al. (1997); Louisy et al. (1994)
Computational modeling	Human, animal	Acute or chronic	Pennline and Mulugeta (2014)
Animal suspension	Animal	Chronic	Gaignier et al. (2014)
Rotating wall vessel (RWV)	Cells or tissue cultures	Chronic	Tsao et al. (1992); Becker and Souza (2013)

**Table 3:** Experimental conditions employed in partial and microgravity investigations

Gravitational context	Gravity condition	Species/specimen
In-flight	Compare 0 G to AG+	Humans Animals Tissues Cells
Earth	Compare 1 G to AG+	Humans Animals Tissues Cells

### Targeted molecular profiling and AG in space

To our knowledge, there is only one in-flight evaluation of artificial gravity on astronauts. This study explored the vestibular responses to linear accelerations, using a human-rated centrifuge chair aboard the Space Shuttle Neurolab mission (STS-90) in 1998. Four payload crewmembers were exposed to in-flight 1 G centripetal acceleration generated by a centrifuge, with two crewmembers also exposed to 0.5 G centrifugation in-flight. The results of this experiment suggested that centrifugal force of 0.5 and 1 G along the subjects’ longitudinal and transversal axis, respectively, was well tolerated by the crew (Clément et al. 2001). For those astronauts who rode the centrifuge 20 min every other day during a 16-day space mission, cardiovascular deconditioning was reduced, while otolith-ocular reflexes were preserved during and after flight (Moore et al. 2005).

To date, there exist only two studies of animals flown in space and subjected to artificial gravity. These were part of the Soviet Cosmos missions conducted in the 1970s. The first animals to be centrifuged in space were flown on the 20-day Cosmos-782 mission in 1975. Fish and turtles were housed in containers and centrifuged at 1 G, during the 20 day 0 G exposure. The center of the containers was placed at 37.5 cm from the center of a platform rotating at 52 rpm. Post-flight behavior and physiology were comparable to animals at 1 G. Turtles centrifuged at levels as low as 0.3 G showed none of the muscle wasting typically associated with microgravity (Ilyin and Parfenov 1979).

A more extensive investigation was conducted during the 19-day Cosmos-936 mission in 1977. Twenty-five unrestrained male Wistar rats were kept in individual cages and placed in a centrifuge with a radius of 32 cm. An artificial gravity level of 1 G was obtained by spinning the centrifuge at 53.5 rpm.

Compared to animals in microgravity that were not exposed to AG, in-flight centrifugation had a protective effect on the musculoskeletal system and myocardium. However, animals exposed to in-flight AG experienced visual, motor, and vestibular adverse effects, including altered equilibrium, righting reflex, and orientation disorders. These effects were presumably due to the magnitude of the gravity gradient and the high rotation rate of the centrifuge (Gurovsky et al. 1980).

It is important to note that neither the single in-flight human AG study conducted in 1998 nor the two in-flight animal AG studies (1975, 1977) sought to analyze molecular markers in urine, blood, or tissues. Thus, we presently have little understanding of the molecular dynamics when AG is applied to humans or animals, during space flight—the most representative of the AG study paradigms. Therefore, we must rely upon surrogate experiments on bacteria, cells, and other biological specimens. These have been flown on the shuttle’s Spacelab, and in the Mir, Salyut, and Skylab space stations. While extensive molecular profiling is still limited, results indicate that microgravity effects at the cellular level can be substantially reduced by AG (Clément and Slenzka 2006).

The only active or planned human or animal space-based AG studies of which we are aware are sponsored by the Japan Aerospace Exploration Agency (JAXA) and NASA, respectively. The facility for the JAXA animal AG study is now on board the ISS, with proposed experimentation to be completed in 2016. This is a modified cell biology centrifuge with a relatively short arm (15 cm radius) that can operate at a rotation rate of 77 rpm. Six mice will be exposed to 1 G for 1–6 months, while six other mice will live at 0 G for the same duration (J. Robinson, personal communications, August 25, 2015). The study will have to be closely watched, given that previous work (Cosmos-936) with a short radius centrifuge induced significant side effects from the relatively high rotational speed.

NASA is currently funding the development of a Rodent Centrifuge Facility (RCF), which will accommodate as many as 14 modular rodent cages. These can be customized to house 42 mice or 28 rats. The facility will be designed for providing continuous centrifugal force of 0.16, 0.38, 0.66, and 1 G for a minimum of 30 days of unattended operation, though it will be able to accommodate experiments lasting up to 90 days.

### Targeted molecular profiling and AG on earth

Targeted molecular studies of humans comparing in-flight 0 G to artificial 1 G are difficult, since the technical infrastructure for applying 1 G centrifugation of humans in the microgravity environment of space does not presently exist. So, various G parameters (2 G, 3 G, etc.) have been applied to humans at 1 G earth gravity to examine for molecular variance between the different G conditions. For the purpose of this review, we have limited our examination to selective representative studies of up to 3 G.

Schneider et al. examined 11 participants who underwent 15 min acceleration to 3 Gz. The following stress-associated small molecules *increased* in comparison to controls: Serum cortisol 70 %; plasma ACTH three-fold; prolactin two-fold; epinephrine 70 %; norepinephrine 45 %. In accordance with the increase in stress biomarkers, physical wellbeing was decreased (Schneider et al. 2008).

**Fig. 3:**
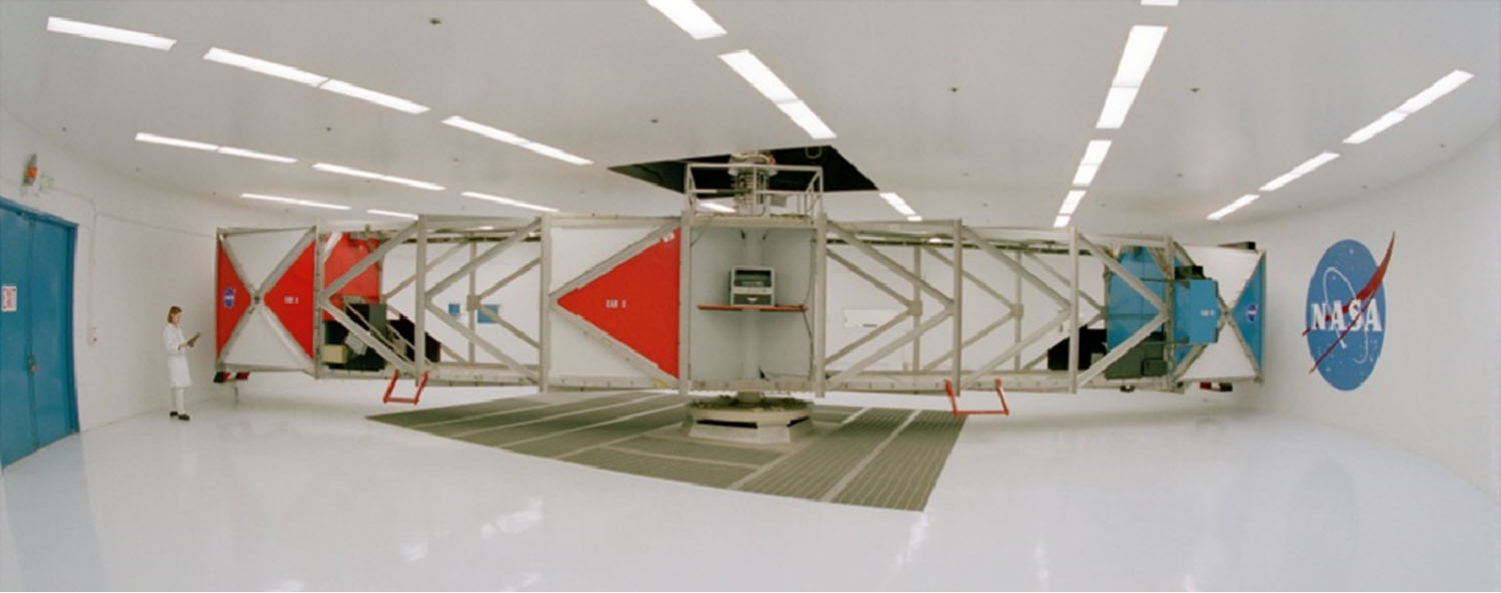
20 G human centrifuge at NASA Ames Research Center. The 20-G Centrifuge is capable of producing forces up to 20 times that of terrestrial gravity, though it is human rated to 12.5 G. The maximum g-level attainable is dependent upon the mass of the specific payload. Mounted on the 58-foot diameter centrifuge are three enclosed cabs. The universal rail mounting system enables novel experiment configurations to be placed at any radius along the arm, allowing comparison of radius, acceleration gradients, or coriolis effects. It is rated to 50 RPM. (Image: NASA)

As one of only two such investigations of which we’re aware, our group conducted a feasibility study on the NASA Ames 20-G Long Arm Human Centrifuge (Fig. 3) to determine how well humans can maintain orthostatic tolerance during and after prolonged exposures to hypergravity. A secondary objective was to examine the targeted urinary metabolome for selected markers of oxidative stress. Four healthy adult men, 20–34 years old, were tested during sustained 22 hour exposures in the centrifuge at constant gravitational loads of 1.0 G (baseline) and 1.25 G. One subject underwent additional 22-h exposure at 1.5 G (Schmidt 2003; Toscano 2013).

Physiological data were collected continuously, which included G-tolerance tests and measures of blood pressure, impedance cardiography, skin conductance, skin temperature, respiration rate, and heart rate. Self-reports of mood and symptoms, along with a cognitive performance test were assessed at 2–4 h intervals. Serial urine samples were collected and assayed for 8-epi-PGF2α (F2-isoprostane), 8-hydroxy-2-deoxyguanosine, malondialdehyde, 4-hydroxynonenal, lipid hydroperoxides, and aqueous hydroperoxides.

 Participants A, B, and C each completed the 1.25 G exposure. One participant experienced syncope after 14 h at 1.25 G, though he continued with the study. This small pilot study revealed that 22-h continuous centrifugation does not produce significant, linear changes in oxidative markers at 1.0 and 1.25 G. However, 22-h continuous centrifugation of a single human subject at 1.5 G resulted in significant nausea and mood deterioration. These symptoms were concomitant with a sharp rise in 8-epi-PGF2α, followed by a sudden drop. 8-epi-PGF2α is an oxidative product of arachidonic acid and a stereoisomer of the smooth muscle agonist PGF2α (an enzymatic product of arachidonic acid).

This finding led us to a new hypothesis related to the potential role of 8-epi-PGF2α in eliciting gastrointestinal smooth muscle symptoms in various conditions of hyperoxia and hypoxia encountered in extreme environments (acute mountain sickness, high altitude flight, etc.). The study was terminated after the 1.5 G exposure, due to potentially high risk of medical injury to the participants.

## Moving from targeted to untargeted discovery in AG studies

As noted, targeted studies generally involve the pre-selection of a small number of analytes. For the purpose of illustration, two analytes important to muscle metabolism in space are briefly explored here; myostatin and PGC-1α.

Myostatin is a member of the TGFβ (transforming growth factor beta) protein family that inhibits myogenesis (muscle differentiation and growth). In rats exposed to 17 days of microgravity on NeuroLab (NASA STS-90), significant increases in myostatin were coupled with pronounced muscle wasting (Lalani et al. 2000). Specifically, the myostatin/beta-actin mRNA ratios were higher in the muscles of the NeuroLab rats compared with the control rats on earth, as follows: 5.0-fold in tibialis, 3.0-fold in biceps, 1.9-fold in quadriceps, and 2.2-fold in gastrocnemius. This implicates elevated myostatin in the muscle wasting of microgravity and space flight.

PGC-1α (peroxisome proliferator activated receptor co-activator 1α) is referred to as the master regulator of mitochondrial biogenesis. In addition to co-activating PPARγ, PGC-1α is also a co-regulator of NRFs and ERRα. It thereby regulates mitochondrial biogenesis and mitochondrial numbers (Scarpulla 2011; Scarpulla et al. 2012). Secretion of PGC-1α has been examined on earth during 5 weeks of unilateral limb unloading in adult humans, wherein those in the unloading group showed a decrease in PGC-1α expression of 36 %, compared with controls **(**Fernandez-Gonzalo et al. 2014). This would seem to have potentially significant implications for the mitochondrial content of muscle in the space flight environment.

### The human powered centrifuge example

From this and a fairly robust body of evidence, myostatin and PGC-1α would, then, seem among the useful markers upon which to base testing of a future AG protocol. In general, one can envision a testing paradigm of a *human powered centrifuge* (or any other AG solution) either in the 1 G environment of earth or the microgravity environment of space. In this example, one would test various loading conditions using the human powered centrifuge for their effects on myostatin and PGC-1α. This would be done in order to establish protocols optimized to those biomarkers, along with attention to the optimized musculoskeletal phenotype. One could extend this line of investigation to a range of molecules and phenotypes that affect the immune system, nervous system, cardiovascular system, and so on.

It is also important to note that, in studying the gravitational ‘input’ of the HPC, attention must be given to the atmosphere in which the gravitational exposure takes place (the exposome). Earth based centrifugation studies are likely to be conducted under ambient O_2_ and CO_2_ conditions, unless designed otherwise. The DRMs on the ISS, planned spacecraft, and currently envisioned space suits call for varying composition of these gases. For instance, the official operating range for CO_2_ levels on the ISS is 0.25–2.10 % (Law et al. 2010).

According to NASA, symptom reports have emerged over the years that suggest an increased sensitivity to CO_2_ in microgravity and that lower limits than these (Earth normal is 0.03 %), may be needed to prevent potential CO_2_ toxicity, cognitive impairment, physical performance changes, and symptoms. Indeed, performance decrements may turn out to be more sensitive indicators than symptoms. For instance, mean raw scores for seven of nine scales of decision-making performance (basic activity, applied activity, task orientation, initiative, information usage, breadth of approach, and basic strategy) have shown to consistently decline with increasing CO_2_ concentrations in humans (overall *p*-values <0.001; Satish et al. 2012).

Therefore, in conducting AG experiments, the habitation atmospheres one is likely to encounter in space warrant serious consideration. Failure to address the habitation atmosphere composition (exposome) during earth-based AG experiments may lead to AG solutions that are not optimally reflective of the space environment.

## Untargeted molecular assessment (omics) in space and on earth

One can use cardiovascular, neuromuscular, ocular, and vestibular physiology to further illustrate this dynamic. Each of these vital areas of aerospace medicine utilizes a broad suite of technologies to characterize the physiological, morphological, and behavioral responses to different space (or test) conditions. This has led to enormous advances in our understanding. With full respect to these advances, we must also acknowledge that complex molecular networks lay beneath these types of physiologic outputs being measured and, further, that the underlying molecular events are partially responsible for the genesis of such physiological, morphological, and behavioral dynamics. Omics-based analyses represent a new suite of tools that permit comprehensive examination of this underlying molecular landscape in relation to these phenotypes.

We have previously asserted that omics application in human space flight provides unique advantages in terms of identification of novel patterns of variance, novel identification of new countermeasure targets, and acceleration of discovery timelines (Schmidt and Goodwin 2013). We suggest here that the addition of omics profiling to artificial gravity experiments represents a novel way to more quickly understand key areas of variance in the molecular landscape that is influenced by the variable artificial and partial gravity conditions being tested.

Use of omics methods will help investigators detect precise changes across broad molecular networks, as various G-loading paradigms are applied. This will be useful in detecting off-target, or unanticipated effects of the different gravity paradigms applied to humans or animals. These data-rich approaches can be used to generate new hypotheses and to develop new potential targets of investigation. Moreover, data from such omics experiments can be correlated with all other forms of physiologic phenotyping. Insights gained from these approaches may eventually be used to inform countermeasure development or refine the deployment of existing countermeasures.

### Untargeted molecular profiling and AG in space

Presently, there are no in-flight human or animal AG studies that have used untargeted methods to examine the molecular landscape. There are, however, limited cell-based analyses that have been conducted. Disruption of immune vigilance is an important challenge to space travel and one representative cell-based, in-flight omics study sheds light on potential human susceptibility to microbes in space. In one of the only studies of its kind, Chang et al. studied donor supplied (n = 4) human T cells, which were stimulated with Con A and anti-CD28 on board the International Space Station (Chang et al. 2013).

An on-board centrifuge was used to generate a 1 G simultaneous control to isolate the effects of microgravity from other variables of spaceflight. In the microgravity group, 617 genes were differentially expressed compared to on-orbit 1 G controls. Forty-seven of these genes had 2-fold or lower expression in microgravity compared to the on-orbit 1 G control. cREL gene targets were significantly inhibited in microgravity. The Rel/NF-κB transcription factor family is comprised of five subunits: cRel, RelA (p65), NF-κB-1 (p50), NF-κB-2 (p52), and RelB. Analysis of gene connectivity indicated that this TNF-linked pathway is a major early downstream effector pathway inhibited in microgravity and may lead to ineffective host defenses against infectious pathogens during spaceflight.

### Untargeted molecular profiling and AG on earth

**Fig. 4:**
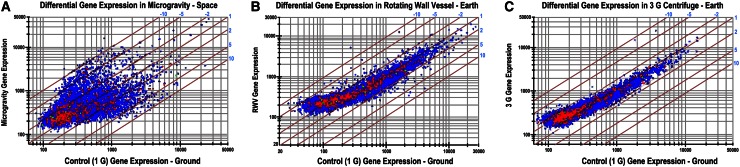
Variant gene expression profiles in three gravity conditions. The response of human embryonic kidney cells to three gravity conditions is shown. Gene expression profiles are distinctly different, with the microgravity condition showing the greatest variance. Upregulated genes are shown in *red*, while down-regulated genes are shown in *blue*. This represents the first findings from microgravity and bioreactor experiments showing genome variance, as cells are transitioned to the reduced gravity environment of space (Hammond et al. 1999, 2000) (Color figure online)

It has previously been shown that human embryonic kidney cells undergo distinctly different gene expression changes under (1) earth gravity (1 G), (2) earth gravity plus centrifugation at 3 G, and (3) reduced gravity in space (0 G or µG) (Kaysen et al. 1999); (Hammond et al. 1999, 2000) (Fig. 4). This fundamental discovery supports the general premise that the biological changes that occur in response to different gravity conditions are broad and complex. These changes in gene expression are typically accompanied by a changing protein (Pastushkova et al. 2013) and small molecule metabolite landscape, which can be further characterized by proteomics and metabolomics.

An untargeted omics approach to hippocampal gene expression has been explored in hypergravity on earth. Del Signore et al. tested the mRNA level of about 5000 genes in the hippocampus of mice subjected to 1.09 G (1 G) or to 1.85 G (2 G) for five repeated 1-h daily rotations in a centrifuge (Del Signore et al. 2004; Mandillo et al. 2003). Data were compared with those obtained for mice stationary at 1 G (C, Control). Roughly 200 genes were affected by rotation and/or rotation + hypergravity. Almost all the genes affected by rotation + hypergravity were up-regulated, with only five being down-regulated. The genes most influenced by the variant gravity conditions code for proteins active across a range of cellular functions (cell cycle and apoptosis, signal transduction, neuronal structure/function, DNA/RNA metabolism, protein processing, intermediary metabolism, cytoskeleton and motility). Six affected genes were directly or indirectly involved in synaptic transmission and plasticity (ESTs moderately similar to thymosin beta-10, syndet, inhibin beta E and Ngfi-A binding protein 2, proSAAS, neuroblastoma ras oncogene).

A single human study further illustrates the potential value of omics approaches to varied gravity conditions. In this case, 12 human subjects were assigned to receive 1 G, 2 G, and 3 G exposures on a *continuous* (SAHC1) basis, with 6 of those subjects being further exposed to *intermittent* (SAHC2) artificial gravity forces, following a recovery period. The continuous centrifugation protocol (SAHC1) consisted of three phases, where the first and the third phase were comprised of application of different G-levels (1 G, 2 G, then 3 G) for 2 min each. During the second (interim) phase, subjects were stressed with continuous exercise (Andjelic 2010).

The intermittent centrifugation protocol (SAHC2) was also divided into three phases, wherein the first and the third phase were identical to those of SAHC1. However, in contrast to SAHC1, exercise stress (second phase) was applied to test subjects intermittently over 30 min by alternating centrifugation and rest every 3 min, which resulted in a total of 5 cycles.

**Fig. 5:**
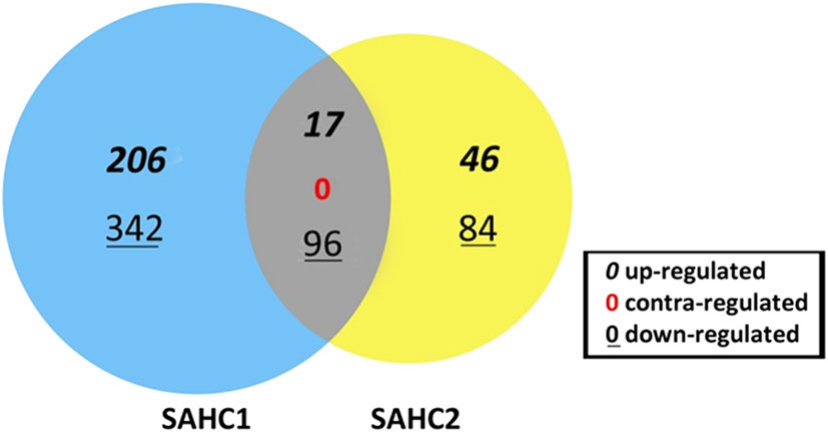
Venn diagram of transcriptome response to continuous and intermittent centrifugation in humans. Up-regulated and down-regulated genes are shown for the continuous (SAHC1; continuous centrifugation protocol; n = 12) and intermittent (SAHC2; intermittent centrifugation protocol; n = 6) centrifugation protocols. p ≤ 0.05 and FC ≥ 1.5 (Adapted from Andjelic 2010) (Color figure online)

The investigators used DNA microarrays to compare each subject’s transcriptome (the entirety of transcribed RNA) prior to and subsequent to centrifugation at each of the three G conditions (30,950 results). The SAHC1 transcript list contained 661 differentially expressed genes of the *continuous* protocol of which 223 (206 + 17) were significantly up-regulated and 483 (342 + 96) were significantly down-regulated (Fig. 5). The SAHC2 transcript list contained 243 differentially expressed genes of the *intermittent* protocol, of which 63 (46 + 17) were significantly up-regulated and 180 (84 + 96) were significantly down-regulated. There were 17 common genes in both protocols that were significantly up-regulated and 96 common genes that were significantly down-regulated. The criteria for significance were established at p ≤ 0.05 and FC ≥ 1.5 (FC, fold change).

Transcriptome data was further subjected to pathway analysis and to biological function analysis. For instance, one significantly up-regulated class of proteins (*p* = 0.0332) was the class of *antibacterial response protein*, to which 5 genes were attributed. Another up-regulated class of proteins (*actin and actin related proteins*) was significantly affected (*p* = 0.0345), with 2 genes identified. By further illustration, 2 significantly upregulated genes (*p* = 0.0437) were found commonly associated with two distinct receptor mediated signaling pathways: Voltage-dependent L-type calcium channel subunit beta-2 (CACNB2) and 1-phosphatidylinositol 4,5-bisphosphate phosphodiesterase beta-4 (PLCB4). Both are involved in oxytocin and serotonin 5HT2 receptor mediated signaling pathways (Andjelic 2010).

This progression from transcriptome analysis, to biological function analysis, to pathway analysis illustrates how one can glean novel insights into the biological change associated with the different AG paradigms, which can be used to further refine all forward experimental design and countermeasure development efforts.

We undertook a pilot study (NASA HRI-246) to determine the practicality of using the NASA Ames 20 G long arm (27.36 ft., 8.8 m) and short arm (8.36 ft., 2.5 m) centrifuge to quantify effects of different G levels, radii, and their resulting gravity gradients on body fluid distribution, cardiovascular regulatory responses, and the effect of lower limb exercise (Fig. 3). We also evaluated two plasma specimens (baseline and endpoint) from three subjects under three separate hypergravity conditions to evaluate experimental design elements related to conducting metabolomics analysis on a very small subject population (Schmidt and Howarth 2007; Howarth 2014).

The conditions included, Condition A: Short Radius (1–1.25 G) No Exercise (Legs Extended); Condition C: Long Radius (1–1.25 G) No Exercise (Legs Extended); Condition H: Long Radius (1–2.5 G) Legs Up (Variable Exercise). The metabolomics portion of the study generated roughly 16,000 molecular features, which were evaluated using principal component analysis. The only condition showing any baseline to endpoint trend was in the three subjects, during the Long Radius-Variable Exercise paradigm, though these results must be taken with caution due to small subject numbers. The study clearly illustrated the unique challenges of small subject and large variable numbers encountered in space-related omics studies, and highlighted the special consideration that must be given to experimental design in such research.

## Omics sub-disciplines applied to the AG environment

Each of the primary omics disciplines below has the capacity to reveal unique patterns of molecular variance in AG experiments, which can inform countermeasure parameters. Note that the examples below reflect single target molecules, but they are used to illustrate the types of markers that can be generated, as part of untargeted analyses applied within any given AG context.

### Genomics

Assess the DNA exome, including single nucleotide polymorphisms, copy number variants, insertions, and deletions. SNPs can inform investigators about susceptibility to various G conditions and aid in determination of whether there is an optimum or limiting genotype (single genes or clusters of genes) for a given G condition. Example: the brain-derived neurotrophic factor (BDNF) Val66Met variant may influence cognitive function, psychological coping, or neuronal repair (Zuccato and Cattaneo 2009; Soliman et al. 2010).

### Epigenomics

Transcriptomic regulating factors not empirically coded in the genomic sequence. Epigenome patterns under various G-conditions may aid in identification of gene regulating effects that impact the AG response (Casey et al. 2015). Example: methylation of the BDNF promoter may be detrimental to neural function in astronauts under the stress of space (Kang et al. 2013), while methylation of the PGC-1α promoter may affect muscle, bone, brain, heart, and other tissues in space (Barrès et al. 2009).

### Transcriptomics

Assessment of gene expression through RNA transcripts. Gene expression patterns under various G-conditions may aid in detecting different patterns of change over time. This may yield new potential assessment targets and lead to new countermeasure targets. Example: varied expression of IL-1β or TNFα may result in altered immune function in space (Crucian et al. 2014).

### Proteomics

Assessment of protein expression. Protein expression patterns under various G-conditions may aid in optimizing the AG parameters. Example: elevated myostatin protein is associated with muscle wasting and contributes to the atrophic effects of glucocorticoids on skeletal muscle (Schakman et al. 2013).

### Metabolomics

Assessment of the small molecule pool (below 1500 amu). Metabolite patterns may reveal changes across metabolic networks under varied G conditions. Example: altered lipidome may provide broad information related to signaling cascades, dietary influences on the AG response, inflammatory pathways, and putative cell membrane composition (Montoliu et al. 2014).

### Exposomics

Assessment of the exposome. The exposome is the sum of environmental exposures and the response to those exposures (Wild 2012; Miller and Jones 2014; Miller 2014). In space flight, an important aspect of the exposome is the percent composition and partial pressure of gases that comprise the habitation and exploration atmospheres, such as CO_2_ and O_2_. The full complement of integrated omics can be used to describe the human response to these exposures. Example: elevated habitation carbon dioxide has been associated with decrements in multiple scales of decision making, such as task orientation, focused activity, initiative, applied activity, and information usage (Satish et al. 2012).

### Metallomics

Assessment of the entirety of metals and metalloids within a cell or tissue type (Szpunar 2005; Mounicou et al. 2009). Broadly speaking, metallomics is the study of the metallome, interactions, and functional connections of metal ions and other metal species with genes, proteins, metabolites, and other biomolecules in biological systems (Lobinski et al. 2010). A specific example of how metallomics may be deployed as an ‘experimental artificial gravity sensor’ is outlined below.

## Omics analysis as an “experimental artificial gravity sensor”

As noted, determining the appropriate artificial gravity condition is among the more daunting challenges facing AG investigators. One aspect of this challenge derives from selecting the ideal combination of phenotypic elements on which to base the decision of which gravity condition is ideal for astronauts.

Since loss of muscle is one of the key reasons for deployment of artificial gravity, muscle represents a useful tissue for determination of the appropriate AG condition. It is important to recognize, however, that muscle turns over slowly and sensitive surrogate molecular measures of muscle may be additionally useful.

Magnesium is easily the most widespread metal ion cofactor found in enzymatic systems (Cowan 2002). This is principally related to the ability of Mg^2+^ to form stable complexes with phosphate-containing species in physiologic systems, such as muscle (Sissi and Palumbo 2009).

Studies from the International Space Station (ISS) have previously shown that urinary Mg levels were 44 % lower after landing than before launch (*p* < 0.001). Specifically, 55 % of ISS crew members had Mg concentrations lower than the low end of the clinical range (3.0 mmol/d) (Smith et al. 2005a, b). Moreover, after six months in space, there is a loss of Mg reservoirs, with 35 % loss in some leg muscles (Fitts et al. 2010). This is noteworthy, as muscle and bone are the principal reservoirs for Mg in the human body, accounting for 27 % and 53 % of total body Mg, respectively (Keller 1991).

A recent paper (Smith and Zwart 2015) endeavored to address selected aspects of the Mg and space flight question. While it did not attempt to address the impact of Mg on DNA (see below), it did highlight the need for substantially more research on the trajectory of tissue magnesium stores in astronauts on long duration missions. The space flight-induced loss of Mg from its reservoirs renders magnesium a potentially useful surrogate marker that is related to the muscle response in altered gravity. One can envision close monitoring of the magnesium trajectory during AG experiments as a specific and, potentially, sensitive measure of the muscle response to gravitational loads. Beyond Mg, one can further envision assessment of the metallome, as a broader measure of the response to varied AG conditions. In this context, the metallome may be viewed as one potential ‘experimental artificial gravity sensor’ that may inform the refined assessment of any given AG parameter under investigation.

### Gravity, radiation, and the metallome

When considering the artificial gravity question, it is reasonable that we also consider an important, converging variable simultaneously encountered in the space environment—radiation. While changing muscle Mg dynamics (and, perhaps, the metallome in general) in altered gravity is potentially revealing, the changing Mg pool may also impact how astronauts respond to radiation. To better understand the implications of this, it is important to review the central role of Mg in DNA repair and view this in the context of space radiation exposure.

We have previously raised concerns that magnesium loss associated with space flight may reduce DNA repair at a time when efficient DNA repair is most needed—in the high radiation environment of space (Schmidt and Goodwin 2013). This is based on three fundamental roles of magnesium in DNA repair, including (1) as a catalytic center of numerous DNA repair enzymes, (2) as the key trace element that stabilizes all ADP and ATP molecules (ATP being required for DNA repair), and (3) as a core element in ATP synthase.

With specific reference to ATP, Magnesium bound to ATP is the sole biologically active form of ATP found in humans (wherein Mg is bound to the phosphate groups of ADP and ATP, as MgATP^2−^). Moreover, Mg^2+^ is a central ion in ATP synthase, binding to phosphate in the catalytic F_1_ moiety of ATP synthase.

Magnesium is an essential cofactor in almost all enzymatic systems involved in DNA processing, with a stabilizing effect on DNA and chromatin structure (Hartwig 2001). In general, magnesium cations bind to DNA and reduce the negative charge density, thereby stabilizing the structure of DNA (Anastassopoulou and Theophanides 2002). Magnesium is an essential cofactor for enzyme systems involving DNA repair, such as mismatch repair, base excision repair, nucleotide excision repair, and double-strand break repair (Hartwig 2001; Nishino and Morikawa 2002).

For example, Apurinic/apyrimidinic endonuclease 1 (APE1) is a powerful enzyme that absolutely requires Mg^2+^ and is the major AP endonuclease in mammalian cells (Manvilla et al. 2013). Previous studies have shown that the robust APE1 activity can be fully suppressed by rigorous chelation of Mg^2+^ (Erzberger and Wilson, 1999; Maher and Bloom 2007), suggesting that Mg^2+^ is essential for APE1 activity and DNA repair. In general, APE1 hydrolyzes the phosphodiester bond at abasic sites, producing 5′-deoxyribose phosphate (dRP) and the 3′-OH primer needed for repair synthesis. APE1 also mediates the repair of single-strand and double-strand breaks by removing blocking groups, such as fragmented sugar moieties (Manvilla et al. 2013).

DNA ligase I (LIG1) catalyzes the ligation of single-strand breaks to complete DNA replication and repair. The energy of ATP is used to form a new phosphodiester bond in DNA via a reaction mechanism that involves three distinct Mg^2+^-dependent chemical steps. At saturating concentrations of ATP and Mg^2+^ ions, the three chemical steps occur at similar rates, and the efficiency of DNA ligation is high. However, under conditions of reduced Mg^2+^, the nick-sealing step becomes rate-limiting, which may significantly compromise DNA replication and repair (Taylor et al. 2011).

Topoisomerases are DNA backbone-processing enzymes. They produce a topological change in a DNA chain by the unwinding or the supercoiling of the double helix (Champoux 2001). This releases the torsional strain imposed by DNA processing (Schoeffler and Berger 2008). Topoisomerases frequently require cofactors for their full catalytic activity. ATP (as MgATP^2−^) regulates the conformational changes required for enzyme action through binding and hydrolysis processes. Thus, divalent Mg^2+^ is required for catalytic and for structural functions, while also forming complexes with ATP. In both topoisomerase IA and topoisomerases II, the Mg^2+^ ions are tightly anchored to the protein backbone through four carboxyl side chain residues (Asp, Glu) (Sissi and Palumbo 2009).

Beyond the specific examination of magnesium and the general examination of the metallome, broader omics methods may be used to survey other molecular features that may serve as ‘experimental artificial gravity sensors.’ This is the landscape on which unpredictable patterns of variance associated with a given gravity condition may be revealed.

A final illustration of this potential can be found in the work of Caiozzo et al. (2009). Male participants (29 ± 3 year) were subjected to 21 days of 6° head-down tilt bed rest (BR group; n = 7) and compared to those on 21 days of bed rest, plus daily exposure to 1 h bouts of 2.5 Gz (as measured at the feet; AG group; n = 8).

Twenty-one days of bed rest produced a 20 % reduction in muscle fiber cross-sectional area in the soleus muscle, whereas it was unchanged in the AG group. This was accompanied by a trend of elevated mRNA levels of myostatin (*p* = 0.09) and atrogin (*p* = 0.12) in the BR group, consistent with a muscle catabolic state. In contrast, AG was effective in maintaining muscle structure and function in plantar flexors.

While AG resulted in positive phenotypic changes in muscle structure and function, it was not sufficient to prevent losses in slow-to-fast isoform myosin heavy chain (MHC) mRNA isoform transitions and losses in total MHC mRNA levels. Bed rest produced significant reductions (~35 %) in the total MHC mRNA levels in both the soleus and the vastus lateralis muscles. However, the actin mRNA levels were unaffected in either muscle, leading the authors to suggest that the transcriptional regulation of myosin is much more sensitive to loading state than is actin (Caiozzo et al. 2009).

Such findings make a compelling case that broad omics analysis of the molecular landscape may yield more sensitive and early indicators of the biological response to AG than the standard physiologic, morphologic, and behavioral phenotype that is typically measured. At minimum, omics analysis can be expected to provide additional molecular features and greater precision in determining the optimal application of AG.

### Hypothetical AG metabolomics experiment in space

**Fig. 6:**
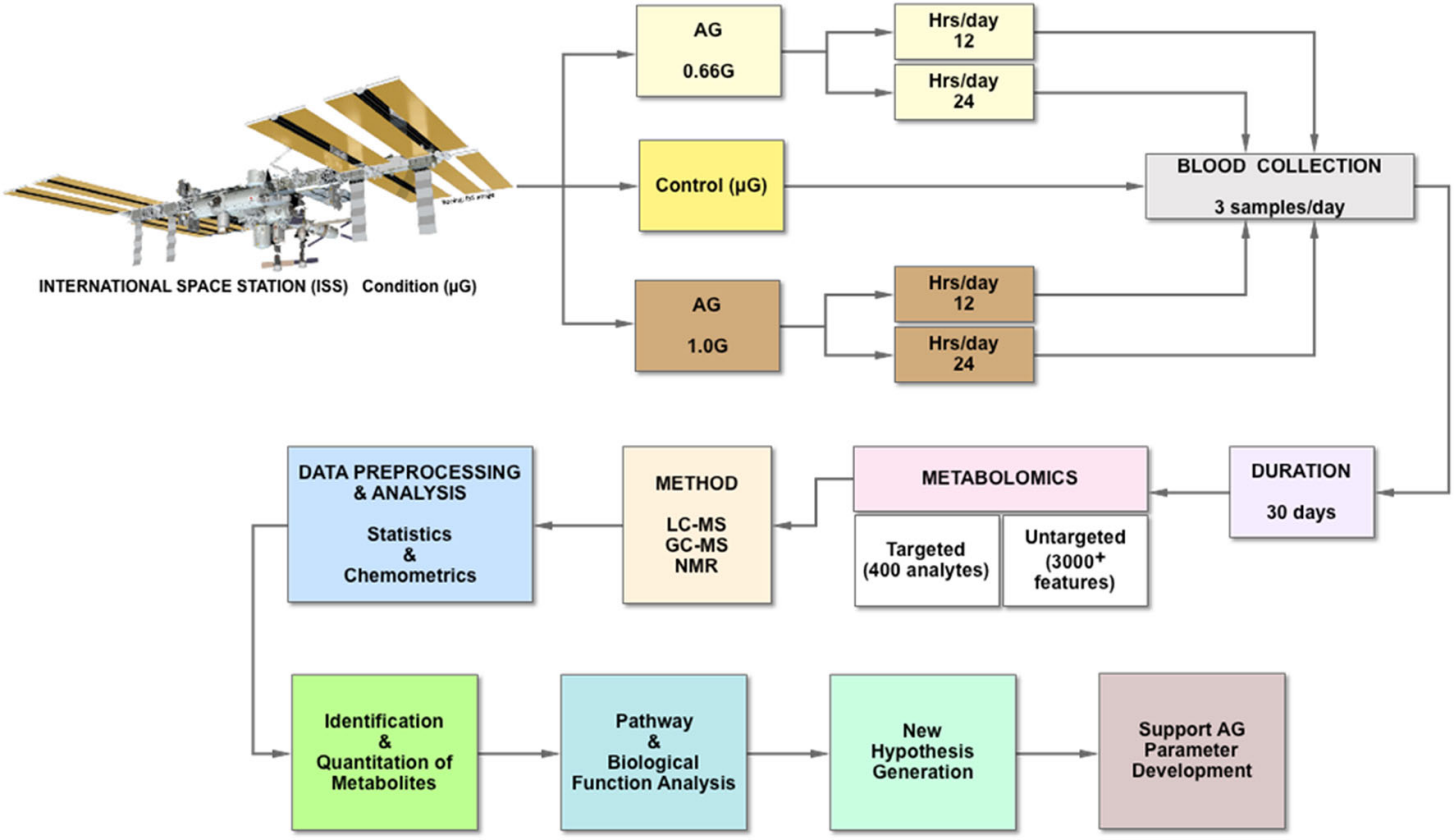
Hypothetical design of an AG metabolomics experiment in space, using the rodent centrifuge facility

The Rodent Centrifuge Facility being deployed on the ISS by NASA can be used to illustrate the use of metabolomics in studying artificial gravity in space (Fig. 6). In this hypothetical study design, animals would be exposed to centrifugation at 0.66 G and 1.0 G (though, under a real experimental scenario, one might also include 0.16 G (Lunar) and 0.38 G (Mars) AG conditions). Duration would be explored by exposing groups to 12 and 24 h of centrifugation each day. These animals would be compared with microgravity controls (µG).

Because of the typical small animal numbers used in such studies, serial measures are recommended in order to develop longitudinal trajectories. These paradigms would be compared over 30 days. Both targeted and untargeted metabolomics would be used, in order to capture known and exploratory analytes.

Analytical platforms, such as LC–MS–MS, GC–MS–MS, and NMR would be employed to identify and quantitate the different molecular forms. Both univariate and multivariate statistics would be used in order to detect variance, determine significance, and identify pathways of interest. This would form the foundation of biological function analysis. A similar model can be used for human AG experimentation in space or on earth, as technology permits.

## Future considerations

The Artificial Gravity Working Group and the affiliated scientific community can benefit from various investigators and teams with know-how in specific omics disciplines. Moreover, various omics-related societies, such as the Metabolomics Society (and others), have recommended standards related to some of the unique experimental issues involved in gathering omics data. This ranges from addressing challenges in experimental design to those of statistical approaches (Dunn et al. 2011; Broadhurst and Kell 2006) that warrant consideration in order to optimize the potential for meaningful discovery involving small subject and large variable numbers.

Our general enthusiasm for collaboration intersects with a conundrum, which derives from a distressed people versus time relationship. The AG research community tasked with asking and answering the seminal AG questions is relatively small. Moreover, these studies are dependent upon access to centrifugation that can be applied to humans and other mammals, under Earth or space conditions. The time lines are also very short for upcoming human missions that include the Moon and Mars. It is perhaps more accurate to say that there is an unusual urgency to this matter, which can be partially addressed by the convergence of investigator teams from omics disciplines with those of the artificial gravity discipline.

Given the paucity of molecular data from space flight AG experiments and the need for more robust molecular data from earth-based AG studies, one could argue that we must do everything we can to gather the greatest depth and breadth of molecular data possible—something that can be aided by omics experiments. Moreover, many AG experiments are unusually difficult to design and execute. Using only targeted physiologic, behavioral, or morphological phenotyping does not take full advantage of the considerable effort waged to develop this rare and complex experimental condition.

We invite the scientific community to consider the following path forward for all future AG experiments, regardless of the experimental condition. First, we recommend designing all artificial gravity experiments in such a way that appropriate specimens for omics investigations can be captured. The design of such experiments could be done in collaboration with the omics community in order to minimize experimental variance, optimize detection of true biological variance, and avoid false discoveries. Second, we suggest investigators biobank these specimens according to current best practices, either for immediate analysis or as archival specimens for future analysis.

Such an approach would be expected to provide more robust data sets, valuable in refining ideal mission AG parameters. This approach may also yield unexpected countermeasure targets, leading to adjunct solutions that could be used to augment any chosen AG parameters. In our view, this represents a unique opportunity to significantly advance one of the key elements supporting the sustained presence of humans in space.
